# Synthetic Kavalactone Analogues with Increased Potency and Selective Anthelmintic Activity against Larvae of *Haemonchus contortus* In Vitro

**DOI:** 10.3390/molecules25082004

**Published:** 2020-04-24

**Authors:** H.M.P. Dilrukshi Herath, Aya C. Taki, Nghi Nguyen, José Garcia-Bustos, Andreas Hofmann, Tao Wang, Guangxu Ma, Bill C.H. Chang, Abdul Jabbar, Brad E. Sleebs, Robin B. Gasser

**Affiliations:** 1 Faculty of Veterinary and Agricultural Sciences, The University of Melbourne, Parkville, Victoria 3010, Australiaaya.taki@unimelb.edu.au (A.C.T.); Jose.GarciaB@unimelb.edu.au (J.G.-B.); a.hofmann@structuralchemistry.org (A.H.); tao.wang1@unimelb.edu.au (T.W.); guangxu.ma@unimelb.edu.au (G.M.); jabbara@unimelb.edu.au (A.J.); 2 Walter and Eliza Hall Institute of Medical Research, Parkville, Victoria 3052, Australia; nguyen.n@wehi.edu.au; 3 Faculty of Medicine, Dentistry and Health Sciences, The University of Melbourne, Parkville, Victoria 3010, Australia

**Keywords:** kavalactone, synthetic analogues, anthelmintic, in vitro-activity, *Haemonchus contortus*

## Abstract

Kava extract, an aqueous rhizome emulsion of the plant *Piper methysticum*, has been used for centuries by Pacific Islanders as a ceremonial beverage, and has been sold as an anxiolytic agent for some decades. Kavalactones are a major constituent of kava extract. In a previous investigation, we had identified three kavalactones that inhibit larval development of *Haemonchus contortus* in an in vitro-bioassay. In the present study, we synthesized two kavalactones, desmethoxyyangonin and yangonin, as well as 17 analogues thereof, and evaluated their anthelmintic activities using the same bioassay as employed previously. Structure activity relationship (SAR) studies showed that a 4-substituent on the pendant aryl ring was required for activity. In particular, compounds with 4-trifluoromethoxy, 4-difluoromethoxy, 4-phenoxy, and 4-*N*-morpholine substitutions had anthelmintic activities (IC_50_ values in the range of 1.9 to 8.9 µM) that were greater than either of the parent natural products—desmethoxyyangonin (IC_50_ of 37.1 µM) and yangonin (IC_50_ of 15.0 µM). The synthesized analogues did not exhibit toxicity on HepG2 human hepatoma cells in vitro at concentrations of up to 40 µM. These findings confirm the previously-identified kavalactone scaffold as a promising chemotype for new anthelmintics and provide a basis for a detailed SAR investigation focused on developing a novel anthelmintic agent.

## 1. Introduction

Kava extract is an aqueous emulsion that is prepared from the plant *Piper methysticum*. It has been used for centuries by Pacific Islanders as an inebriant beverage in ritual and cultural ceremonies [1,2]. Since the 1990s, the commercial extract has been ‘popularized’ as an anxiolytic agent in countries including the USA, Australia, New Zealand, and Germany, although its use has been restricted in some countries due to concerns regarding possible, rare hepatotoxicity associated with consumption [3]. Kavalactones, also called kavapyrones, are lipophilic pyrones, which usually concentrate in the roots of the plant and are responsible for the anxiolytic activity of the kava extract [4]. To date, 18 such kavalactones have been identified, and six of them (i.e., kavain, dihydrokavain, methysticin, dihydromethysticin, desmethoxyyangonin, and yangonin) have been reported to be responsible for ~96% of the pharmacological activity of this extract [5,6].

In a recent effort to discover anthelmintic candidates from natural sources [7], we identified, for the first time, an enriched root extract from *P. methysticum*, collected from Papua New Guinea, with activity against one of the most important parasitic nematodes of livestock animals—*Haemonchus contortus*. Subsequent bioassay-guided fractionation of this extract yielded three kavalactones (i.e., dihydrokavain, desmethoxyyangonin, and yangonin) that inhibited the development of exsheathed third-stage larvae (xL3s) to fourth-stage larvae (L4s) [7]. While dihydrokavain had a limited potency at inhibiting L4 development (IC_50_ of 207.6 µM), both desmethoxyyangonin (IC_50_ of 31.7 µM) and yangonin (IC_50_ of 23.7 µM) were moderately potent inhibitors of larval development. Interestingly, each of these compounds induced a lethal ‘evisceration’ (Evi) phenotype in treated larvae [7]. Using one representative from the identified kavalactone panel, desmethoxyyangonin, we also showed that exposure to the compound for 30 h was sufficient to induce the Evi phenotype in *H. contortus* (see [7]). All three kavalactones (i.e., dihydrokavain, desmethoxyyangonin, and yangonin) had limited toxicity on MCF10A, non-tumorigenic breast epithelial cells in an in vitro-cell proliferation assay [7]. Together with knowledge of the drug-like features of kavalactones, these findings suggested that the kavalactone (α-pyrone) scaffold has potential for ‘lead’ identification in the quest to develop new nematocides. Therefore, we undertook a structure-activity relationship (SAR) investigation of this natural product scaffold, with the aim of synthesizing analogues with improved efficacy and pharmacokinetic profiles, and with a view toward identifying potential ‘lead’ candidates for future anthelmintic drug development. 

## 2. Results and Discussion

**Figure 1:**
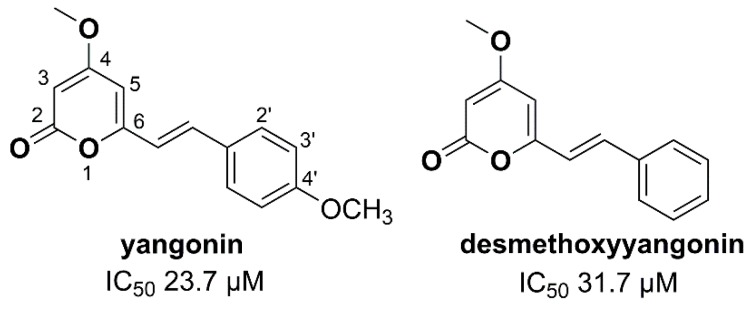
Structures of the natural products, yangonin and desmethoxyyangonin, and their activities against *Haemonchus contortus* in the larval development assay (see [7]).

Initially, desmethoxyyangonin and yangonin were synthesized in-house and evaluated for their anthelmintic activity. These kavalactones had IC_50_ values (for larval development-inhibition) in a similar range to those purchased and evaluated in a previous study [7], confirming the activity of the synthesized compounds. The IC_50_ values obtained with desmethoxyyangonin **1** were 31.7 µM (purchased) and 37.1 µM (synthesized) for larval development-inhibition, and the values for yangonin **2** were 23.7 µM (purchased) and 15.0 µM (synthesised) (Table 1; Figure 1). The methoxy-substitution at the 4-position of the pendant phenyl ring is the only difference between desmethoxyyangonin and yangonin, which might be the reason for the observed differences in the IC_50_ values. 

**Figure 2:**
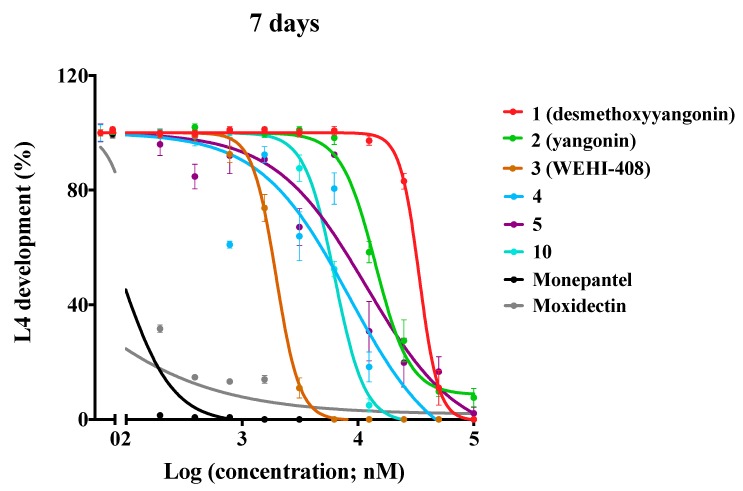
The activity of desmethoxyyangonin, yangonin and selected analogues (**3**, **4**, **5,** and **10**) against *Haemonchus contortus* in the larval development assay (conducted over 7 days). Dose-response curves for the two parent kavalactones (**1** and **2**) and four analogues (**3**, **4**, **5** and **10**) for developmental inhibition in comparison to monepantel or moxidectin. Information on compounds is given in Table 1.

To investigate the SAR of the kavalactone framework, we synthesized 17 analogues to explore the effect of the pendant aryl ring-substitution on anthelmintic activity. Seven of the synthesized analogues had IC_50_ values of < 20 µM for L4 development-inhibition; four of them had IC_50_ values of < 10 µM. Specifically, the potencies of the analogues **3** (WEHI-408), **4**, **5,** and **10**, with IC_50_ values ranging from 1.9 to 8.9 µM, were greater than those of their parent kavalactones desmethoxyyangonin **1** and yangonin **2** (Table 1; Figure 2). 

The SAR derived from the bioassay data (Table 1) showed that analogues **14** and **15**, with 2-methoxy or 3-methoxy substitution on the phenyl ring, did not possess significant anthelmintic effects (IC_50_ values of 61.9 µM and > 100 µM, respectively). In contrast to the activity of **2**, bearing a 4-methoxy substitution, the data showed that methoxy substitutions at the 2- or 3-position were not tolerated. Analogues **16**–**18** with 3,4-methylenedioxy, 3-cyano, or 3-carboxylate substitutions on the phenyl ring were also inactive, further confirming that substitution at the 3-position is not tolerated. 

A set of analogues with varying substitutions at the 4-position of the phenyl ring showed that analogues **3**–**5** with 4-(trifluoromethoxy), 4-(difluoromethoxy) and 4-phenoxy substituents significantly increased anthelmintic activity (IC_50_ values of 1.9, 8.9, and 5.2 µM, respectively) compared with the 4-methoxy parent compound **2** (Table 1). Analogues **6**–**8** with 4-chloro, 4-methyl, and 4-trifluoromethyl substitutions all had comparable activities (IC_50_ values of 12.4, 12.6, and 10.3 µM, respectively) to yangonin **2**, suggesting that non-polar functionalities with electron neutral or withdrawing properties and with less steric volume than a methoxy group were tolerated. In contrast, analogues **9**, **12,** and **13** with the polar groups dimethylamino, cyano, and carboxylate at the 4-position were inactive at the highest concentration tested. Interestingly, the analogue **10** with *N*-morpholine in the 4-position exhibited an IC_50_ of 6.4 µM, but the 4-*N*-piperazine analogue **11** was inactive, suggesting that there is a balance between the polarity and lipophilicity of functionalities tolerated at the 4-position. Notably, the activity of analogues **5** and **10** with phenoxy or morpholine groups in the 4-position suggests that these groups with a large steric volume could be orientated towards solvent space, or alternatively, be accommodated in a suitable cavity within the presumed protein-target of the kavalactones. The addition of a morpholine group to the 4-position had the added benefit of likely enhancing the aqueous solubility of the kavalactones. Additional solubility improvements could accrue from the addition of an endocyclic nitrogen; however, the only synthetically tractable position on the pendant aryl ring is the 3-position, and analogue **19** was shown to be inactive at the highest concentration tested.

Summarizing the activity data, the anthelmintic potency of the parent kavalactones **1** and **2** was significantly increased by minor substitutions on the 4-position of the phenyl-ring, without modifying the α-pyrone scaffold (Table 1). Importantly, all analogues with a significant inhibitory effect induced an Evi phenotype (cf. [7]), suggesting that these analogues have a mode of action that is the same or similar to their respective parent kavalactone.

All analogues assessed for inhibitory activity against *H. contortus* were also tested against human HepG2 hepatoma cells in vitro (Table 1), using CellTiter-Glo^®^ to measure ATP concentration as a metabolic marker for cell numbers. The data showed that none of the compounds inhibited cell growth at the highest concentration tested (IC_50_ > 40 µM), with analogue **3** (WEHI-408) having the highest selectivity index (> 20-fold) against *H. contortus*. Overall, this investigation showed that four of 17 analogues synthesized (i.e., **3**, **4**, **5,** and **10**) had enhanced potency against and selectivity for *H. contortus*, without evidence of significant in vitro-toxicity on hepatoma cells. Clearly, this increased anthelmintic activity and lack of apparent in vitro-toxicity of analogues **3**, **4**, **5,** and **10**, all bearing relatively minor structural modifications, indicate a pathway for future work on the pyrone scaffold toward novel anthelmintic leads.

Given that the development of new anthelmintics is critical to counteract widespread resistance problems in nematodes of livestock animals, the current investigation—enabled by our previous study of kavalactones [7]—offers guidance for developing the α-pyrone scaffold into an anthelmintic. Although different synthetic kavalactones and analogues have been reported in the literature [8,9,10,11], none of them had been tested against a parasitic nematode of animals. The target/s of natural kavalactones or synthetic analogues has/have not yet been identified in nematodes, but some targets, including γ-aminobutyric acid (GABA) receptors, voltage-gated ion channels, monoamine oxidase, and the arachidonate cascade, have been proposed for mammals [12,13,14,15,16,17,18,19]. Orthologues of these proteins in *H. contortus* and related parasites should be investigated as possible targets responsible for the anthelmintic activity of the kavalactones reported here. Available genomic, transcriptomic, and proteomic data sets (see [20,21,22,23]) would provide a foundation for such an extension. 

## 3. Materials and Methods 

### 3.1. Chemical Synthesis of Kavalactones and Analogues

Nineteen compounds were synthesized. In brief, desmethoxyyangonin **1**, yangonin **2,** and all the 4-methoxypyrone derivatives (**3**–**19**) were synthesized by condensation of the appropriate aryl aldehyde with 4-methoxy-6-methyl-2H-pyran-2-one under basic conditions, following a previously published method [24] (Supplementary file 1, Scheme S1). Synthesis protocols and compound characterization are given in the Supplementary file 1.

### 3.2. Procurement of H. contortus

The Haecon-5 strain of *H. contortus* was produced in experimental sheep [21,25], according to animal ethics guidelines of the University of Melbourne, Australia (permit no.1413429). The feces were collected from the infected sheep and incubated at 27 °C for 1 week, allowing first-stage larvae to emerge and develop to third-stage larvae (L3s) [25]. L3s were collected and stored at 10 °C until used in experiments; xL3s were prepared by treating L3s with 0.15% *v/v* sodium hypochlorite (NaClO) and incubated for 20 min at 38 °C, and subsequently washed five times with sterile physiological saline (pH 7.0). These xL3s were immediately incubated at 38 °C in sterile Luria Bertani (LB) broth supplemented with 100 IU/ml of penicillin, 100 µg/ml of streptomycin, and 0.25 mg/ml of amphotericin B (fungizone, antibiotic—antimycotic; cat. no. 15240-062; Gibco, Thermo Fisher Scientific, Waltham, MA, USA); this supplemented medium was designated as LB*.

### 3.3. Assessment of Potency of Synthesized Compounds at Inhibiting Larval Development of H. contortus

In our previous study [7], larval development-inhibition results guided the fractionation of the active extract from *P. methysticum*, and the findings were consistent with subsequent potency assessments of the purified kavalactones. Therefore, the developmental inhibition assay for *H. contortus* was used to determine the activity/potency of the kavalactone analogues synthesized in the present study. In brief, all synthesized compounds as well as monepantel (Zolvix, Elanco Animal Health, Australia) and moxidectin (Cydectin, Virbac, France) (two positive controls) were individually tested for inhibition of larval development in *H. contortus* using an 18-point, two-fold dilution series, commencing at 100 µM. Compound solutions (in dimethyl sulfoxide [DMSO] plus LB*) were prepared and added to 96-well flat bottom plates to achieve the desired final concentrations in a final volume of 100 µl, and xL3s (as described in Section 3.2) were dispensed into individual wells at a density of 300 worms per well (in 50 µl of LB*). Matched concentrations of DMSO (0.25% in LB*) were used as negative (untreated) controls. The plates were incubated (10% *v*/*v* CO_2_) at 38 °C for 7 days [25]. Larvae in each well were fixed with 50 µl of 1% iodine at 7 days, and the number of xL3s that developed to L4s in each well was counted by light microscopic examination at 20× magnification [25]. L4s were differentiated from xL3s by their well-developed mouth and pharynx characteristics [26]. The assay was repeated three times on different days using three replicates in each assay. The half-maximum inhibitory concentration (IC_50_) of each compound was determined by fitting the log_10_-transformed concentration to a variable slope four-parameter model using the GraphPad Prism (v. 8.3.1) software (GraphPad Software Inc., San Diego, CA, USA).

### 3.4. Assessment of Cytotoxicity and Selectivity of Synthesized Compounds

Synthesized compounds were tested for their toxicity on HepG2 human hepatoma cells, as described previously [27]. In brief, compounds were tested in a 10-point, two-fold dilution series, commencing at 40 µM. The cells were cultured in Dulbecco’s modified Eagle’s medium (DMEM) supplemented with 10% fetal bovine serum (FBS) and incubated at 37 °C with 5% *v/v* CO_2_. The cells were seeded into 384-well plates at a density of 1 × 10^3^ per well and exposed to compounds for 48 h. The toxicity was determined by CellTiter-Glo^®^ cell viability assay (Promega, USA), which assessed the number of viable cells based on the quantity of ATP present. Matched percentages of etoposide and DMSO were used as positive and negative (untreated) controls, respectively. The EC_50_ values were determined as described in Section 3.3, and the selectivity index was calculated by dividing the EC_50_ value for HepG2 cells by the IC_50_ value for *H. contortus*. Bortezomib, which has an EC_50_ of 0.01 µM, was used as the reference control compound.

## Figures and Tables

**Table 1:** The activities of synthesized kavalactone analogues against *Haemonchus contortus* in the larval development assay and HepG2 human hepatoma cells in vitro. A comparison of half maximum inhibitory concentration (IC_50_) values of the synthesized compounds with those of monepantel and moxidectin, expressed as mean IC_50_ ± standard error of the mean (SEM).

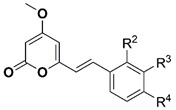					
Compound	R^2^	R^3^	R^4^	Inhibition of Larval Development in *H. Contortus* IC_50_ (µM) ± SEM	HepG2 EC_50_ (µM)
**1 (desmethoxyyangonin)**	H	H	H	37.1 ± 3.1	> 40
**2 (yangonin)**	H	H	OCH_3_	15.0 ± 3.0	> 40
**3 (WEHI-408)**	H	H	OCF_3_	1.9 ± 0.1	> 40
**4**	H	H	OCHF_2_	8.9 ± 0.3	> 40
**5**	H	H	OPh	5.2 ± 2.2	> 40
**6**	H	H	Cl	12.4 ± 3.5	> 40
**7**	H	H	Me	12.6 ± 2.1	> 40
**8**	H	H	CF_3_	10.3 ± 1.2	> 40
**9**	H	H	N(CH_3_)_2_	> 50	> 40
**10**	H	H	*N*-morpholine	6.4 ± 0.2	> 40
**11**	H	H	*N*-piperazine	> 100	>40
**12**	H	H	CO_2_Me	> 100	> 40
**13**	H	H	CN	> 100	> 40
**14**	OCH_3_	H	H	> 100	>40
**15**	H	OCH_3_	H	61.9 ± 7.3	> 40
**16**	H	-OCH_2_O-		> 100	> 40
**17**	H	CN	H	> 100	> 40
**18**	H	CO_2_Me	H	> 100	> 40
**19**	H	[N]	OCH_3_	> 100	> 40
**Monepantel**				0.07 ± 0.01	nd
**Moxidectin**				0.02 ± 0.00	nd

nd = not determined.

## Supplementary Materials

The following are available online, Chemistry methods, Scheme S1. General synthetic pathway for kavalactone analogues. References [9,24,28,29] are cited in the supplementary materials
