# Supplementary material for: Synthetic Kavalactone Analogues with Increased Potency and Selective Anthelmintic Activity against Larvae of Haemonchus contortus In Vitro
Source: Molecules. 2020 Apr 24;25(8):2004. doi: 10.3390/molecules25082004 (PMC7221733; doi:10.3390/molecules25082004)
Supplement: Supplementary file 1 [file molecules-25-02004-s001.pdf]

molecules

Supplementary file 1

Synthetic Kavalactone Analogues with Increased Potency and Selective Anthelmintic Activity Against Larvae of *Haemonchus contortus* In Vitro

H.M.P. Dilrukshi Herath [1,†], Aya C. Taki [1,†], Nghi Nguyen [2,3], José Garcia-Bustos [1], Andreas Hofmann [1], Tao Wang [1], Guangxu Ma [1], Bill C.H. Chang [1], Abdul Jabbar [1], Brad E. Sleebs [2,3,*] and Robin B. Gasser [1,*]

1 Faculty of Veterinary and Agricultural Sciences, The University of Melbourne, Parkville, Victoria 3010, Australia; dili.herath@unimelb.edu.au (H.M.P.D.H.); aya.taki@unimelb.edu.au (A.C.T.); Jose.GarciaB@unimelb.edu.au (J.G.-B.); a.hofmann@structuralchemistry.org (A.H.); tao.wang1@unimelb.edu.au (T.W.); guangxu.ma@unimelb.edu.au (G.M.); bill.chang@yourgene-health.com (B.C.H.C.); jabbara@unimelb.edu.au (A.J.)
2 Walter and Eliza Hall Institute of Medical Research, Parkville, Victoria 3052, Australia; nguyen.n@wehi.edu.au (N.N.)
3 Faculty of Medicine, Dentistry and Health Sciences, The University of Melbourne, Parkville, Victoria 3010, Australia
† These authors contributed equally to this work
* Correspondence: robinbg@unimelb.edu.au (R.B.G.); sleebs@wehi.edu.au (B.E.S.)

Chemistry Methods

General Chemistry Procedures

All non-aqueous reactions were performed under an atmosphere of nitrogen, unless otherwise specified. Commercially available reagents were used without further purification. Analytical thin-layer chromatography was performed on Merck silica gel $60F^{254}$ aluminum-backed plates and visualized by fluorescence quenching under UV light or by $KMnO_4$ staining. Flash chromatography was performed with silica gel 60 (particle size 0.040-0.063 μm). NMR spectra were recorded on a Bruker Avance DRX 300 with the solvents indicated (^{1}H NMR at 300 MHz). Chemical shifts are reported in ppm on the δ scale and referenced to the appropriate solvent peak. LCMS conditions used to assess the purity of compounds were as follows: column—XBridge TM C18 5 μm 4.6 × 100 mm, injection volume 10 μL, gradient: 10%–100% B over 10 min (solvent A: water 0.1% formic acid; solvent B: AcCN 0.1% formic acid), flow rate: 1.5 mL/min, detection: 100–600 nm. Unless otherwise stated, all compounds were shown to be >95% pure by this method.

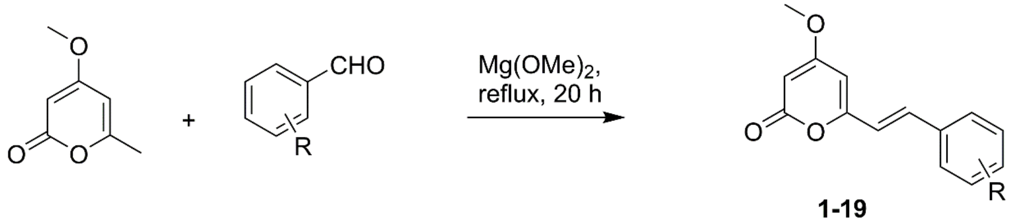

Scheme 1. General synthetic pathway for kavalactone analogues.

General Procedure for Preparation of Kavalactone Analogues 1–19.

Kavalactone analogues were prepared according to the previously reported method [24]. A mixture of 4-methoxy-6-methyl-pyran-2-one (25 mg, 0.178 mmol) and appropriate aldehyde (0.178 mmol) in dimethoxymagnesium solution (60% in 2 mL methanol) was heated to reflux under a nitrogen atmosphere for 20 h. After the reaction was completed, the mixture was concentrated under reduced pressure. The crude product was purified by flash column-chromatography (0%–40% EtOAc/cHexane) to give the title compound.

The ^{1}H NMR data for compounds **1**, **2**, **9**, **14**, **15,** and **16** were consistently in agreement with those previously reported in the literature [9,24,28,29].

(*E*)-4-Methoxy-6-styryl-2*H*-pyran-2-one 1 (desmethoxyyangonin). Yield 22%; Off-white solid. ^{1}H NMR (300 MHz, $CDCl_3$) δ 7.59–7.49 (m, 3H), 7.45–7.33 (m, 3H), 6.61 (d, *J* = 16.0, 0.4 Hz, 1H), 5.97 (dt, *J* = 2.2, 0.5 Hz, 1H), 5.52 (d, *J* = 2.2 Hz, 1H), 3.85 (d, *J* = 0.2 Hz, 3H). ^{13}C NMR (75 MHz, $CDCl_3$) δ 171.1, 164.0, 158.7, 135.8, 135.2, 129.5, 128.9, 127.5, 118.6, 101.4, 88.9, 56.0. LC-MS (ESI) *m*/*z* 229.0 $[M + H]^+$.

(*E*)-4-Methoxy-6-(4-methoxystyryl)-2*H*-pyran-2-one 2 (Yangonin). Yield 22%; beige solid**.** ^{1}H NMR (300 MHz, $CDCl_3$) δ 7.55–7.43 (m, 3H), 6.98–6.88 (d, *J* = 6.0 Hz, 2H), 6.48 (d, *J* = 15.9 Hz, 1H), 5.92 (d, *J* = 2.2 Hz, 1H), 5.50 (d, *J* = 2.2 Hz, 1H), 3.86 (s, 3H) 3.85 (s, 3H). LC-MS (ESI) *m*/*z* 259.1 $[M + H]^+$. ^{1}H NMR data agree with literature.[2]

(*E*)-4-Methoxy-6-(4-(trifluoromethoxy)styryl)-2*H*-pyran-2-one 3 (WEHI-408). Yield 15%; colourless solid**.** ^{1}H NMR (300 MHz, $CDCl_3$) δ 7.59–7.44 (m, 3H), 7.25 (d, *J* = 8.3 Hz, 2H), 6.57 (d, *J* = 15.9 Hz, 1H), 5.98 (d, *J* = 2.2 Hz, 1H), 5.54 (d, *J* = 2.2 Hz, 1H), 3.86 (s, 3H). LC-MS (ESI) *m*/*z* 313.2 $[M + H]^+$.

(*E*)-6-(4-(Difluoromethoxy)styryl)-4-methoxy-2*H*-pyran-2-one 4. Yield 14%; beige solid. ^{1}H NMR (300 MHz, DMSO) δ 7.73 (d, *J* = 8.7 Hz, 2H), 7.46 (d, *J* = 57.4 Hz, 1H), 7.31 (s, 1H), 7.22 (d, *J* = 8.6 Hz, 2H), 7.10–6.94 (m, 1H), 6.32 (d, *J* = 2.2 Hz, 1H), 5.66 (d, *J* = 2.2 Hz, 1H), 3.84 (s, 3H). LC-MS (ESI) *m*/*z* 295.0 $[M + H]^+$.

(*E*)-4-Methoxy-6-(4-phenoxystyryl)-2*H*-pyran-2-one 5 Yield 22%; beige solid. ^{1}H NMR (300 MHz, DMSO) δ 7.69 (d, *J* = 8.6 Hz, 2H), 7.54–7.14 (m, 4H), 7.14–6.83 (m, 5H), 6.31 (d, *J* = 2.3 Hz, 1H), 5.66 (d, *J* = 2.3 Hz, 1H), 3.85 (s, 3H). LC-MS (ESI) *m*/*z* 321.0 $[M + H]^+$.

(*E*)-6-(4-Chlorostyryl)-4-methoxy-2*H*-pyran-2-one 6. Yield 14%; beige solid. ^{1}H NMR (300 MHz, DMSO) δ 7.74–7.65 (m, 2H), 7.49 (d, *J* = 8.5 Hz, 2H), 7.33 (d, *J* = 16.3 Hz, 1H), 7.05 (d, *J* = 16.2 Hz, 1H), 6.33 (d, *J* = 2.2 Hz, 1H), 5.68 (d, *J* = 2.2 Hz, 1H), 3.84 (s, 3H). LC-MS (ESI) *m*/*z* 263.0 $[M + H]^+$.

(*E*)-4-Methoxy-6-(4-methylstyryl)-2*H*-pyran-2-one 7. Yield 23%; beige solid**.** ^{1}H NMR (300 MHz, DMSO) δ 7.56 (s, 1H), 7.34 – 7.18 (m, 3H), 6.95 (d, *J* = 16.1 Hz, 1H), 6.30 (d, *J* = 2.2 Hz, 1H), 5.64 (d, *J* = 2.2 Hz, 1H), 3.84 (s, 3H), 2.33 (s, 3H). LC-MS (ESI) *m*/*z* 243.0 $[M + H]^+$. ^{1}H NMR data agree with literature.[4]

(*E*)-4-Methoxy-6-(4-(trifluoromethyl)styryl)-2*H*-pyran-2-one 8. Yield 23%; beige solid. ^{1}H NMR (300 MHz, DMSO) δ 7.88 (d, *J* = 8.1 Hz, 2H), 7.78 (d, *J* = 8.3 Hz, 3H), 7.42 (d, *J* = 16.2 Hz, 2H), 7.19 (d, *J* = 16.2 Hz, 1H), 6.41 (d, *J* = 2.3 Hz, 1H), 5.71 (d, *J* = 2.2 Hz, 1H), 3.86 (s, 3H). LC-MS (ESI) *m*/*z* 297.2 $[M + H]^+$.

(*E*)-6-(4-(Dimethylamino)styryl)-4-methoxy-2*H*-pyran-2-one 9. Yield 6%; yellow solid**.** ^{1}H NMR (300 MHz, $CDCl_3$) δ 7.53 – 7.37 (m, 3H), 6.72 (d, *J* = 8.5 Hz, 2H), 6.40 (d, *J* = 15.8 Hz, 1H), 5.87 (d, *J* = 2.2 Hz, 1H), 5.46 (d, *J* = 2.2 Hz, 1H), 3.84 (s, 3H), 3.04 (s, 6H). LC-MS (ESI) *m*/*z* 272.2 $[M + H]^+$. ^{1}H NMR data agree with literature.[2]

(*E*)-4-Methoxy-6-(4-morpholinostyryl)-2*H*-pyran-2-one 10. Yield 7%; yellow solid. ^{1}H NMR (300 MHz, $CDCl_3$) δ 7.55–7.39 (m, 3H), 6.91 (d, *J* = 8.8 Hz, 2H), 6.52–6.38 (m, 1H), 5.90 (d, *J* = 2.2 Hz, 1H), 5.48 (d, *J* = 2.2 Hz, 1H), 3.93–3.86 (m, 4H), 3.86–3.82 (m, 3H), 3.33–3.20 (m, 4H). LC-MS (ESI) *m*/*z* 314.2 $[M + H]^+$.

(*E*)-4-Methoxy-6-(4-(piperazin-1-yl)styryl)-2*H*-pyran-2-one hydrochloride 11. Yield 20%; yellow solid. ^{1}H NMR (300 MHz, DMSO) δ 8.95 (s, 2H), 7.56 (d, *J* = 8.6 Hz, 2H), 7.26 (d, *J* = 16.0 Hz, 1H), 7.03

(d, *J* = 8.6 Hz, 2H), 6.83 (d, *J* = 16.0 Hz, 1H), 6.24 (d, *J* = 2.2 Hz, 1H), 5.60 (d, *J* = 2.2 Hz, 1H), 3.83 (s, 3H), 3.47 (m, 4H), 3.22 (m, 4H). LC-MS (ESI) *m/z* 313.2 $[M + H]^+$.

(*E*)-Methyl 4-(2-(4-methoxy-2-oxo-2*H*-pyran-6-yl)vinyl)benzoate 12. Yield 9%; beige solid. ^{1}H NMR (300 MHz, $CDCl_3$) δ 8.15–7.98 (m, 1H), 7.63–7.42 (m, 2H), 6.69 (dd, *J* = 16.0, 0.5 Hz, 1H), 3.95 (s, 2H), 3.86 (s, 1H). LC-MS (ESI) *m/z* 287.1 $[M + H]^+$.

(*E*)-4-(2-(4-Methoxy-2-oxo-2*H*-pyran-6-yl)vinyl)benzonitrile 13. Yield 15%; beige solid. ^{1}H NMR (300 MHz, $CDCl_3$) δ 7.69 (d, *J* = 8.4 Hz, 2H), 7.63–7.55 (m, 2H), 7.50 (d, *J* = 16.0 Hz, 1H), 6.68 (dd, *J* = 16.0, 0.4 Hz, 1H), 6.04 (dt, *J* = 2.2, 0.5 Hz, 1H), 5.56 (d, *J* = 2.2 Hz, 1H), 3.87 (s, 3H). LC-MS (ESI) *m/z* 254.2 $[M + H]^+$.

(*E*)-4-Methoxy-6-(2-methoxystyryl)-2*H*-pyran-2-one 14. Yield 20%; beige solid**.** ^{1}H NMR (300 MHz, $CDCl_3$) δ 7.81 (d, *J* = 16.1 Hz, 1H), 7.51 (d, *J* = 7.7 Hz, 1H), 7.33 (d, *J* = 15.4, 7.4 Hz, 1H), 7.03–6.85 (m, 2H), 6.73 (d, *J* = 16.1 Hz, 1H), 5.96 (s, 1H), 5.51 (s, 1H), 3.92 (s, 3H), 3.85 (s, 3H). LC-MS (ESI) *m/z* 259.2 $[M + H]^+$.

(*E*)-4-Methoxy-6-(3-methoxystyryl)-2*H*-pyran-2-one 15. Yield 18%; beige solid. ^{1}H NMR (300 MHz, $CDCl_3$) δ 7.86–7.75 (m, 1H), 7.54–7.45 (m, 1H), 7.33 (ddd, *J* = 8.3, 7.4, 1.7 Hz, 1H), 7.05–6.87 (m, 2H), 6.73 (dd, *J* = 16.2 Hz, 1H), 5.96 (dt, *J* = 2.2, 0.5 Hz, 1H), 5.51 (d, *J* = 2.2 Hz, 1H), 3.92 (d, *J* = 0.3 Hz, 3H), 3.85 (s, 3H). LC-MS (ESI) *m/z* 259.0 $[M + H]^+$.

(*E*)-6-(2-(Benzo[*d*][1,3]dioxol-5-yl)vinyl)-4-methoxy-2*H*-pyran-2-one 16. Yield 13%; beige solid. ^{1}H NMR (300 MHz, $CDCl_3$) δ 7.43 (d, *J* = 15.9 Hz, 1H), 7.06–6.95 (m, 2H), 6.87–6.78 (m, 1H), 6.42 (dd, *J* = 15.8, 0.4 Hz, 1H), 6.02 (s, 2H), 5.92 (dt, *J* = 2.2, 0.5 Hz, 1H), 5.50 (d, *J* = 2.2 Hz, 1H), 3.86–3.83 (m, 3H). LC-MS (ESI) *m/z* 273.2 $[M + H]^+$.

(*E*)-3-(2-(4-Methoxy-2-oxo-2*H*-pyran-6-yl)vinyl)benzonitrile 17. Yield 10%; beige solid. ^{1}H NMR (300 MHz, $CDCl_3$) δ 7.81 (t, *J* = 1.7 Hz, 1H), 7.75 – 7.67 (m, 1H), 7.63 (dt, *J* = 7.7, 1.4 Hz, 1H), 7.56–7.43 (m, 2H), 6.77–6.56 (m, 1H), 6.03 (dt, *J* = 2.2, 0.5 Hz, 1H), 5.56 (d, *J* = 2.2 Hz, 1H), 3.87 (s, 3H). LC-MS (ESI) *m/z* 254.0 $[M + H]^+$.

(*E*)-Methyl 3-(2-(4-methoxy-2-oxo-2*H*-pyran-6-yl)vinyl)benzoate 18. Yield 8%; beige solid**.** ^{1}H NMR (300 MHz, $CDCl_3$) δ 8.27–8.20 (m, 1H), 8.02 (d, *J* = 7.7 Hz, 1H), 7.70 – 7.64 (m, 1H), 7.58–7.45 (m, 2H), 6.69 (d, *J* = 16.0 Hz, 1H), 6.03–5.95 (m, 1H), 5.54 (d, *J* = 2.2 Hz, 1H), 3.97 (d, *J* = 0.5 Hz, 3H), 3.86 (d, *J* = 0.5 Hz, 3H). LC-MS (ESI) *m/z* 287.0 $[M + H]^+$.

(*E*)-4-Methoxy-6-(2-(6-methoxypyridin-3-yl)vinyl)-2*H*-pyran-2-one 19. Yield 10%; beige solid. ^{1}H NMR (300 MHz, DMSO) δ 9.38 (s, 1H), 9.00 (d, *J* = 8.9 Hz, 1H), 8.28 (d, *J* = 16.2 Hz, 1H), 7.98 – 7.73 (m, 2H), 7.21 (s, 1H), 6.60 (s, 1H), 4.81 (d, *J* = 14.9 Hz, 6H). LC-MS (ESI) *m/z* 260.0 $[M + H]^+$.
